# Cement-in-cement stem revision for Vancouver type B periprosthetic femoral fractures after total hip arthroplasty: A 3-year follow-up of 23 cases

**DOI:** 10.3109/17453670903316827

**Published:** 2009-10-01

**Authors:** Toby W Briant-Evans, Darmaraja Veeramootoo, Eleftherios Tsiridis, Matthew J Hubble

**Affiliations:** ^1^Queen Alexandra HospitalPortsmouthUK; ^2^Princess Elizabeth Orthopaedic Centre, Royal Devon and Exeter HospitalExeterUK; ^3^Leeds General InfirmaryLeedsUK

## Abstract

**Background and purpose** Revision surgery for periprosthetic femoral fractures around an unstable cemented femoral stem traditionally requires removal of existing cement. We propose a new technique whereby a well-fixed cement mantle can be retained in cases with simple fractures that can be reduced anatomically when a cemented revision is planned. This technique is well established in femoral stem revision, but not in association with a fracture.

**Patients and methods** We treated 23 Vancouver type B periprosthetic femoral fractures by reducing the fracture and cementing a revision stem into the pre-existing cement mantle, with or without supplementary fixation.

**Results** 3 patients died in the first 6 months for reasons unrelated to surgery. In addition, 1 was too frail to attend follow-up and was therefore excluded from the study, and 1 patient underwent revision surgery for a nonunion. The remaining 18 cases all healed with radiographic union after an average time of 4.4 (2–11) months. There was no sign of loosening or subsidence of the revision stems within the old cement mantle in any of these cases at the most recent follow-up after an average of 3 (0.3–9) years.

**Interpretation** Our results support the use of the cement-in-cement revision in anatomically reducible periprosthetic fractures with a well-preserved pre-existing cement mantle. This technique is particularly useful for the elderly patient and for those who are not fit for prolonged surgical procedures.

## Introduction

The most commonly occurring fractures around a femoral stem are of Vancouver type B (Duncan and Masri 1995, Lindahl et al. 2005). Treatment options include fixation of the fracture alone if the stem is stable (B1 fractures) (Beals and Tower 1996, Ricci et al. 2005, Lindahl et al. 2006). However, if the stem in situ is loose (type B2 and B3), then revision with a long femoral stem is recommended (Dennis et al. 2000, Tsiridis et al. 2003, 2004, Katzer et al. 2006).

There are many advocates for the use of uncemented long stems for revision (Mont and Maar 1994, Springer et al. 2003, Duwelius et al. 2004, O'Shea et al. 2005). Potential disadvantages of this technique, however, include proximal stress shielding, subsidence of the stem, a restriction in weight bearing in the immediate postoperative period, and a requirement for sufficient femoral diaphyseal bone stock to achieve distal fixation. Cemented stem revisions may also be used (McLaughlan et al. 1997, Tsiridis et al. 2004) but it has been suggested that this technique be reserved for those patients who are elderly with poor surrounding bone stock and whose fractures can be reduced anatomically (Beals and Tower 1996, Springer et al. 2003)

Regardless of the choice of replacement stem, revision surgery for periprosthetic fractures around a cemented prosthesis normally necessitates the laborious task of removing the existing cement mantle. An alternative is to retain cement that is well fixed at the cement-bone interface and to use the “cement-in-cement” technique for femoral component revision. To reduce the intraoperative time and complications—particularly in high risk, more elderly patients—we performed cement-in-cement stem revisions, with or without internal fixation, on a series of patients with Vancouver type B periprosthetic fractures. The objectives of this study were to assess the validity of this technique and to evaluate patient outcomes.

## Patients and methods

### Demographics

A review of the hip surgery registry at our unit identified 23 patients (mean age 79 (54–92) years, 15 men) who underwent cement-in-cement revisions for Vancouver type B periprosthetic femoral fractures between September 1995 and March 2005.

1 patient had an American Society of Anaesthesiologists (ASA) score of 1, 13 were ASA 2, 7 were ASA 3, and 2 were ASA 4. There were 3 Vancouver type B1, 17 type B2, and 3 type B3 fractures. 14 fractures involved primary hip replacements and 9 followed previous revision surgery to the femoral stem (of which 5 were second revisions, 3 were third revisions, and 1 was the fourth revision following primary THR). 3 patients underwent simultaneous acetabular revisions: 1 for a mal-aligned cup and 2 for aseptic loosening. The mean time of fracture from original implantation was 6 (0.4–23) years. 22 fractures involved Exeter stems and 1 involved a Charnley stem. The original cement type was known in 11 cases, all being Simplex. The remaining 12 prostheses were not implanted in our unit and the type of cement was not known.

### Inclusion criteria

Patients were selected for cement-in-cement revision by the treating surgeon on the grounds of their age, comorbidities, fracture configuration, and the radiographic and intraoperative appearance of the bone-cement interface. Most patients were elderly or had multiple comorbidities, requiring a short surgical procedure. The 3 B1 fractures were of transverse or short oblique patterns. These were considered to be unstable fractures that would benefit from the added stability of long stem revision, as it has been shown that there is a high failure rate associated with fixation alone of B1 fractures (Lindahl et al. 2006). The B2 and B3 fractures had 2 principle displaced parts without significant comminution (Table 1). The bone-cement interface in all fractures was judged radiographically and intraoperatively to be stable in all 7 Gruen zones, the cement remaining well fixed to the fracture fragments at the cement-bone interface, even though the prosthesis was now usually loose at the prosthesis-cement interface and the cement mantle by definition fractured in at least one place.

**Table 1.** Fracture configuration in 23 patients

	B1	B2	B3
Spiral	0	16	1
Short oblique	1	0	2
Transverse	2	1	0

### Surgical technique

Following an extended posterior approach to the hip and stem removal, it was common to find some loose, comminuted fragments of cement proximally. These were removed and well-fixed cement was left attached to the fracture fragments but debulked as necessary with a high-speed burr or power reamers to allow the subsequent insertion of a larger, longer stem. This was used to bypass the fracture site in 20 of the 23 cases. In the remaining 3 cases, a standard-length stem was used and a lateral plate and/or cortical strut allograft was used to bridge the fracture site (Table 2). In cases where a long stem was used, the fracture site was bypassed by the revision stem by a mean ratio of 2.5 (1.0–4.7) ipsilateral cortical diameters.

**Table 2.** Revision methods and fixation in 23 patients

	B1	B2	B3	Total
Circlage wires or cables alone	0	10	2	12
Strut graf	1	0	0	1
Cable plate	2	7	1	10
Autograft	1	3	3	7
Long stem revision **^a^**	3	13	3	20
Short stem revision **^a^**	0	4	0	4
**^a^** Length of stem refers to whether the stem bypasses the fracture site by at least 1 cortical diameter (long), or not (short).				

A cement restrictor was inserted distally, with a temporary transfemoral wire placed distal to this to limit migration of the plug during cement pressurization. The fracture was then reduced and stabilized with circlage wires or cables. Additional plates or strut graft were used in 11 of the 23 cases to help stabilize the femur. Autograft was applied to the fracture site in 7 cases to promote fracture union. The revision stem was then cemented into the remaining existing cement mantle using third-generation techniques with Simplex cement. Stem insertion was earlier (within the first 2–3 min) than in routine primary surgery.

It was common for small amounts of cement to escape from gaps in the fracture site due to the high pressures generated during stem insertion. Rather than attempting to occlude such escape, this was allowed so as to limit the escape to this area alone; attempts to seal such vents risk causing the cement to be forced out of the fracture site at other additional areas, which might impair fracture union. Such escaped cement was then removed before wound closure.

### Assessment

Patients were reviewed clinically and radiographically at 6 weeks, 3 months, 6 months, and then annually. Outcomes were assessed in terms of complications, time to union, and function. Union was determined radiographically by the presence of callus bridging the fracture on 2 radiographic views (Tsiridis et al. 2004).

## Results

On average, patients were discharged home or to rehabilitation units from hospital 11 (5–28) days postoperatively. The mean time to partial weight bearing in these patients was 3.6 (1–13) days. The mean time to full weight bearing was 31 (3–120) days.

3 patients died during the first 6 postoperative months, before union could be recorded adequately, and they were thus excluded from the analysis. 1 patient, a 90-year-old man with multiple comorbidities, was seen at 6 weeks but was not subsequently reviewed as he was too infirm to travel from his nursing home. He was reported to have had no problems with his revision until his death 2.5 years later, but as he was not assessed radiographically he was also excluded from our analysis. The remaining 19 fractures in 19 patients were followed up for a mean period of 3 (0.3–9.3) years.

### Complications

1 nonunion occurred, leading to a plate fracturing at the unhealed fracture site 7 months after revision. This patient had extruded cement removed from the fracture site at the time of revision. In addition, the revision stem was not bypassing the most distal fracture line, leading to mechanical failure of the construct. This stem was successfully revised again using the cement-in-cement technique, to a longer Exeter prosthesis. The fracture united and has been problem-free for 3 more years.

### Fracture healing and stem performance

Of the 18 fractures followed up to successful union, the mean time to radiological union was 4.4 (2–11) months (Figure 2).

**Figure 1.**
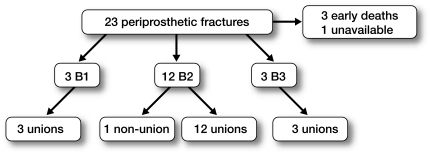
Flow diagram summarizing outcomes.

**Figure 2.**
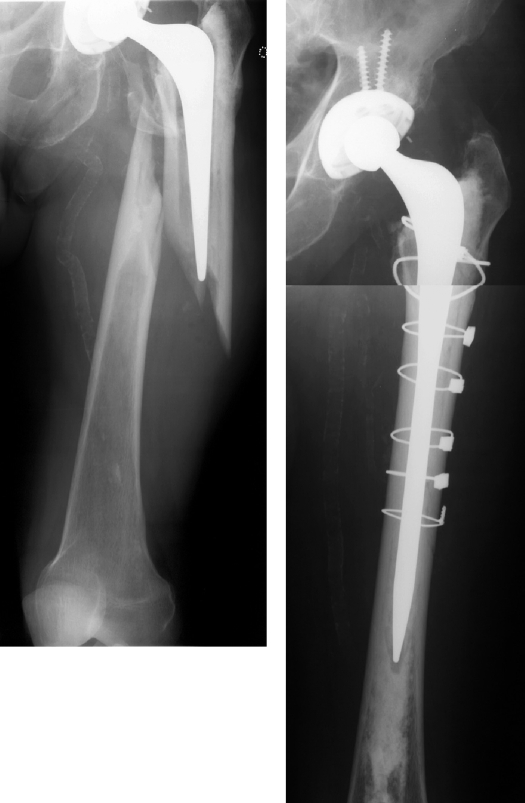
A Vancouver B2 periprosthetic fracture revised using the cement-in-cement revision technique with a long stem and Dall-Miles cables. Note that this was an early case in which a distal cement restrictor was not used.

At the latest radiographic follow-up, there was no evidence of prosthetic loosening or lucency at the new cement-cement interface. The revision polished tapered stems were found to have subsided by an average of 1.0 (0-3.2) mm at a mean follow-up time of 3 years, which is consistent with this design of stem (Timperley et al. 1993, Alfaro-Adrian et al. 2001). There were no cases of clinical infection.

## Discussion

There has been ongoing debate over the use of the cement-in-cement technique for over 30 years, based on laboratory (Greenwald et al. 1978, Rosenstein et al. 1992, Li et al. 1996, Weinrauch et al. 2007) and clinical data (Lieberman et al. 1993, Nelson 2002, Mandziak et al. 2007, Goto et al. 2008). Some reports support its use in revision hip arthroplasty, with no radiographic loosening in 42 cases at 30 months (Quinlan et al. 2006) and no re-revisions for stem loosening in 191 cases at 5–16 year follow-up (Duncan et al. 2009). However, to our knowledge there has been no published work on the use of this technique in periprosthetic fractures.

The largest series of periprosthetic fractures to date is the review by Lindahl et al. (2005) of 688 cases from the Swedish Hip Register. These cases had been revised using various methods; 8% of B1 fractures, 15% of B2 fractures, and 17% of B3 fractures required further operations. Incidences of nonunion and loosening were 6% and 5%, respectively. The mean hospital stay was 21 days. Other authors have found nonunion rates of 5–31% and loosening rates of 10–50% (Beals and Tower 1996, Lewallen and Berry 1998, Springer et al. 2003, Tsiridis et al. 2004, O'Shea et al. 2005).

We found 1 nonunion in 19 patients with a mean follow-up of 3 years, and no cases of loosening. These results, while from a small sample size, are favorable compared to the previous reports.

Concern has been expressed that cement extrusion may inhibit fracture healing (Cooke and Newman 1988, Eschenroeder and Krackow 1998); this was not supported by our study. Although the single incident of nonunion occurred with a case in which cement had leaked from the fracture site intraoperatively, in this case the stem was revised to one that did not bypass the fracture site. This has been found to be a major factor in fracture healing and construct strength, both in the laboratory (Dennis et al. 2000) and in clinical work (Tsiridis et al. 2004), with evidence that revisions using stems that bypass the fracture site are almost 4 times more likely to heal than those with short stems. 2 further patients in our study, both of whom were revised to long stems to bypass the fracture site, had evidence of leaked cement on radiographic follow-up and this did not affect their healing. The mean time to radiographic union of fractures (4.4 months) is similar to those found with other techniques (Tsiridis et al. 2004).

Although it is difficult to make comparisons between the outcomes in our small cohort of 23 patients with the outcomes in more extensive studies (Lindahl et al. 2005), one of the advantages of this technique appears to be the relatively short hospital stay of 11 days. The mean time to full weight bearing of 31 days was skewed by the practice of maintaining partial weight bearing for 120 days in the earlier cases. It is now our routine to start full weight bearing immediately after surgery in these cases.

It is worth noting that all except 1 of the stems revised were Exeter polished taper stems, and all were revised to Exeter stems (long or standard size). The procedure in which the original stem was a Charnley was not complicated by revising to a different stem, as the original mantle is generally reamed up to accommodate a larger revision stem. This particular case showed union at 6 months. It cannot be ascertained from this study whether using alternative revision stems would achieve satisfactory results, although other stem designs have been used with success in published series of cement-in-cement revision hip replacement (Lieberman et al. 1993, Goto et al. 2008).

Although the study was limited by sample size, the results suggest that there is a valid role for the use of the cement-in-cement revision technique for periprosthetic fractures. This method is particularly recommendable for patients who are not suited to lengthy procedures, with simple, reducible fractures associated with a well-fixed cement mantle; it has significant advantages in terms of simplification of operative technique and enhancement of speed of recovery.
